# PPARγ/mTOR signalling: striking the right balance in cartilage homeostasis

**DOI:** 10.1136/annrheumdis-2014-206884

**Published:** 2015-01-14

**Authors:** Francesco Dell'Accio, Joanna Sherwood

**Affiliations:** 1 William Harvey Research Institute, Barts and The London, School of Medicine and Dentistry, Queen Mary, University of London, London, UK; 2 Institute for Experimental Musculoskeletal Medicine, University Hospital Münster, Münster, Germany

**Keywords:** Osteoarthritis, Chondrocytes, Knee Osteoarthritis

Osteoarthritis (OA) is still a highly prevalent and disabling disease for which we do not have a cure. In the last decade, however, the unravelling of molecular mechanisms controlling joint homeostasis, the advances in targeting technologies, and the improvement of animal models allowing the use of mouse genetics has led to the identification of molecular targets that, in animal models,^1–4^ and possibly also in humans^5^ can arrest disease or even revert its course. Such strategies include blockade of extracellular matrix-degrading enzymes,^6^ hypoxia-inducible factor 2α blockade^3^ ^4^ to prevent chondrocyte hypertrophy, the support of parathyroid hormone/parathyroid hormone related protein signalling—an injury-induced homeostatic mechanism affecting both cartilage homeostasis and bone remodelling,^2^ improving bone turnover using strontium ranelate,^5^ and blockade of the filamin-core binding factor interaction thereby supporting chondrocytic differentiation using kartogenin.^7^ Although only strontium ranelate has been tested in the clinic, there is little doubt that the availability of multiple targets will stimulate and instruct the experimentation needed to bridge the gap to the clinic.

In this issue of the journal, Vasheghani *et al*^8^ have demonstrated that PPARγ-driven mTOR (mammalian target of rapamycin) inhibition protects cartilage from experimental OA, at least in part by supporting autophagy,^8^ a process that, by suppressing protein synthesis and enabling the use of cellular components to generate energy, allows cells to escape death in conditions of stress or lack of nutrients.^9^

The authors previously reported that cartilage-specific disruption of the gene encoding for the transcription factor PPARγ results in spontaneous OA in mice.^10^ To ensure that this phenotype was not driven by skeletal dysplasia determined by the absence of PPARγ during development, they generated a new mutant in which PPARγ could be deleted postnatally in chondrocytes upon administration of doxycycline. The mice did not develop spontaneous OA, but became more susceptible to experimental OA induced by destabilisation of the medial meniscus (DMM). The more severe OA phenotype in PPARγ knockout (KO) mice was associated with increased expression of Mmp13, Adamts5, and with a higher number of apoptotic chondrocytes.

The same group^11^ and others^12^ had previously shown that disruption of mTOR, an intracellular kinase promoting cell metabolism, growth, energy usage and differentiation, protected mice from experimental OA with reduced chondrocyte apoptosis, increased autophagy and lower expression of catabolic markers.^11^ ^12^

Therefore they then tested the hypothesis that the worse OA outcome in PPARγ KO mice was due to enhanced mTOR signalling.

mTOR is the catalytic subunit of two distinct molecular complexes, mTORC1 and mTORC2, which are differently regulated, have different downstream effectors (Raptor and Rictor^13^ ^14^) and lead to distinct downstream effects.^9^

Loss of PPARγ resulted in increased expression of mTOR, with a corresponding decrease in autophagy markers in resting conditions or following DMM.

Most strikingly, double KO of mTOR and PPARγ were protected from DMM-induced OA with a phenotype similar to that of the previously published mTOR KO,^11^ thus establishing that, in this context, PPARγ is epistatic to mTOR. This is not trivial, because, in different contexts, and in particular in the regulation of lipid synthesis, mTOR positively regulates the activity of PPARγ.^15^ It is therefore possible that either the epistatic relationship of these two molecules is context dependent, or that PPARγ is activated by mTOR and operates a negative feedback loop to limit mTOR activation.

These data suggest that mTOR is a pathogenic factor in cartilage, which facilitates cell death. A few months earlier, however, Chen and Long^16^ reported that disruption of the mTOR complex 1 (mTORC1) in chondrocytes, through either knockout of mTOR or of the mTORC1-specific mediator Raptor, resulted in a chondrodysplasia characterised by short skeletal elements, with delayed endochondral bone formation. In this context, chondrocyte proliferation was not affected, apoptosis was delayed, not increased, and the phenotype was largely contributed by decreased protein synthesis, decreased extracellular matrix formation, and reduced cell volume, all in keeping with the known functions of mTOR and mTORC1 in particular.

**Figure 1:**
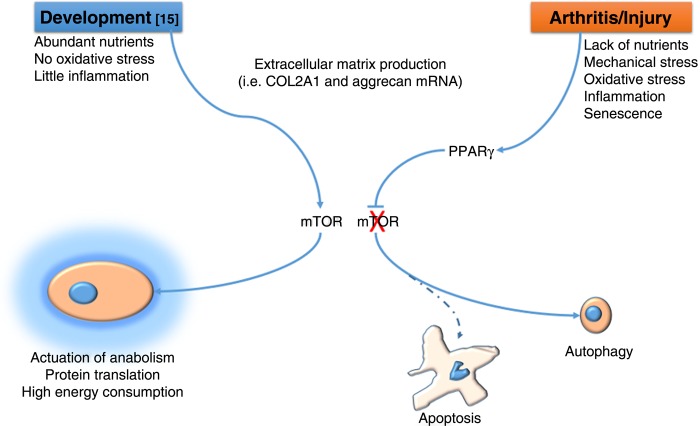
During embryonic skeletal development^16^ mTOR (mammalian target of rapamycin) supports skeletal growth and extracellular matrix production by promoting high energy consumption and protein synthesis . During the early phases of osteoarthritis^8^ PPARγ suppresses mTOR and supports autophagy, which, by reducing protein synthesis and allowing use of cellular components for energy production, affords chondrocytes to escape apoptosis.

How do we reconcile the ‘anabolic’ function of mTOR identified by Chen and Long^16^ in cartilage development with the pathogenic one identified by Vasheghani *et al*^8^ in OA? One could speculate that whereas in embryonic development mTOR is required to support the high energy consumption necessary for skeletal growth and permitted by the abundance of nutrients and absence of injury, in certain phases of OA, particularly in the early phases following injury, rather than trying to compensate to the matrix loss with more anabolic activity, chondrocytes have to ‘lay low’ to escape cell death until the noxious factors are scavenged (figure 1). This hypothesis would also reconcile this literature with other reports showing an anabolic effect of AKT, also involved in mTOR signalling in cartilage.^17^ ^18^

mTOR expression and activity is regulated by a number of signalling pathways, availability of nutrients and metabolism, and therefore PPARγ/mTOR signalling is ideally placed to allow chondrocytes to match what would be desirable following injury (repair, anabolism) with the constrains of a pathologic environment (absence of nutrients,^19^ inflammation, comorbidities, etc).

mTOR is not only an inhibitor of autophagy, but also positively regulates protein synthesis, cell proliferation, differentiation, cell size, and extracellular matrix production,^9^ all processes that in one way or another play a role in OA pathology. Equally, several factors other than PPARγ and inflammation regulate mTOR activity, including availability of nutrients, metabolism and mechanical stress, which are also associated with OA susceptibility and progression.

Apparently in contrast with the well-established role of mTOR in supporting anabolism, protein synthesis and extracellular matrix production including in cartilage,^9^ ^16^ Vasheghani *et al* found that PPARγ KO chondrocytes had increased mTOR but decreased expression of the anabolic markers Aggrecan and COL2A1 mRNA, and PPARγ overexpression resulted in upregulation of COL2A1 and Aggrecan mRNA. Therefore the upregulation of Aggrecan and COL2A1 mRNA may be functions of PPARγ which are independent of mTOR. Alternatively, although not addressed in the article, mTOR suppression of matrix production takes place at the level of protein synthesis,^9^ ^16^ and therefore the upregulation of COL2A1 and Aggrecan mRNA may not translate into protein. Finally, the upregulation of these markers downstream of PPARγ signalling may be the result of an improved survival of differentiated chondrocytes rather than a genuine transcriptional outcome.

The article by Vasheghani *et al*^8^ is also important because both mTOR and PPARγ are pharmacologically targetable with several compounds already available, including PPARγ agonists^20^ ^21^ and several inhibitors of mTOR, the most well-known of which is rapamycin.

So, besides the availability of molecules with a suitable safety profile, tolerability and appropriate pharmacokinetics, what are the gaps in knowledge that we need to fill before clinical translation can be attempted?

The first hurdle is that mTOR and PPARγ are involved in many different processes and their activity is highly context dependent. Within the same cell, mTOR can lead to different biological outcomes depending on its association within mTORC1 and mTORC2 and the subcellular compartmentalisation,^22^ and its activity is influenced by many other signalling pathways including those activated by WNTs, IGF-1 and inflammatory cytokines.^9^ The understanding of how these different signals utilise common components of the intracellular machinery and yet achieve specific outcomes is only starting to bud, but its accomplishment will allow the rational design of specific, safer and more efficacious drugs.

A second issue highlighted by this work is that for effective targeting of adaptive mechanisms we need to be able to identify patients whose disease is driven by such mechanisms at the time of intervention. For example, in the article by Vasheghani *et al*^8^, mTOR was upregulated in PPARγ KO mice also in resting conditions, but the difference in cartilage cellularity was evident only after injury. This is clearly an advantage in terms of safety, because it will not affect healthy cartilage, but also carries its challenges: it might be possible that in different subsets of patients, or in different phases of the disease, for instance when repair is important, shutting down energy consumption and protein synthesis may be undesirable.

A final consideration is that, as mTOR and PPARγ are involved in many metabolic processes, it is highly likely that comorbidities including diabetes and obesity, nutrition and ageing will have profound consequences on the effect of targeting these pathways effectively and safely. A new class of biological readouts and biomarkers are therefore needed that are relevant not necessarily to the disease outcome (eg, cartilage breakdown), but that report on the activity of specific disease mechanisms to predict response to therapy.
